# Nicolau syndrome due to a traumatic finger injury with a needle : a case report with an exclusive photographic documentation

**DOI:** 10.1080/23320885.2020.1805325

**Published:** 2020-08-12

**Authors:** Luis de Almeida Maia, Olga Pereira, Ricardo Marta, Joana Costa, Ana Batista, João David Costa, Fernando Macedo, Jose Fraga, Carlos Vilela

**Affiliations:** a Department of Orthopaedics and Traumatology, Hospital Senhora da Oliveira, Guimarães, Portugal; b Department of Dermatology, Hospital Senhora da Oliveira, Guimarães, Portugal

**Keywords:** Needle, Nicolau syndrome, finger necrosis

## Abstract

**Introduction:**

Nicolau syndrome, also known as livedo-like dermatitis or embolia cutis medicamentosa, is a rare complication usually after intra-muscular or intra-articular injection of various drugs. It is difficult to find photographic documentation of this syndrome from the initial stages due to its rarity and unpredictable evolution.

**Case presentation:**

We report the case of a 54-year-old Portuguese woman who developed Nicolau Syndrome after a traumatic finger injury with a sewing needle. She developed an ulcer and cutaneous necrosis. She was treated with surgical debridement, antibiotic, analgesics and sterile dressings. The ulcer healed completely within 18 weeks with scarring.

**Conclusions:**

Although Nicolau syndrome develops very rarely, it is an important cause for morbidity. It is an iatrogenic condition. The Nicolau Syndrome following a traumatic injury with a needle without drugs, as far as we know, has never been reported in the published literature especially with photographic records from the beginning of the process.

## Introduction

Nicolau syndrome (NS) (Livedoid Dermatitis or Embolia Cutis Medicamentosa) is a rare complication usually after intra-muscular or intra-articular injection of various drugs. We report a case with photographic records from the first day of a 54-year-old Portuguese woman who developed Nicolau Syndrome after a traumatic finger injury with a sewing needle. The NS was firstly described by Freudenthal and Nicolau in 1924 and the first report was in 1925 after the muscular injection of bismuth for the syphilis in the gluteal area [1,2]. The usual characteristics of the injected lesion are pain right after injection, subsequently erythematous lesion, livedoid and hemorrhagic patch, and NS leads to necrosis of skin, adipose and muscle layers [3]. The syndrome has been related to the injection of various drugs, including non-steroidal anti-inflammatory drugs. To our knowledge, there were no literature about NS related after traumatic needle injury without drugs.

## Case presentation

**Figure 1.**
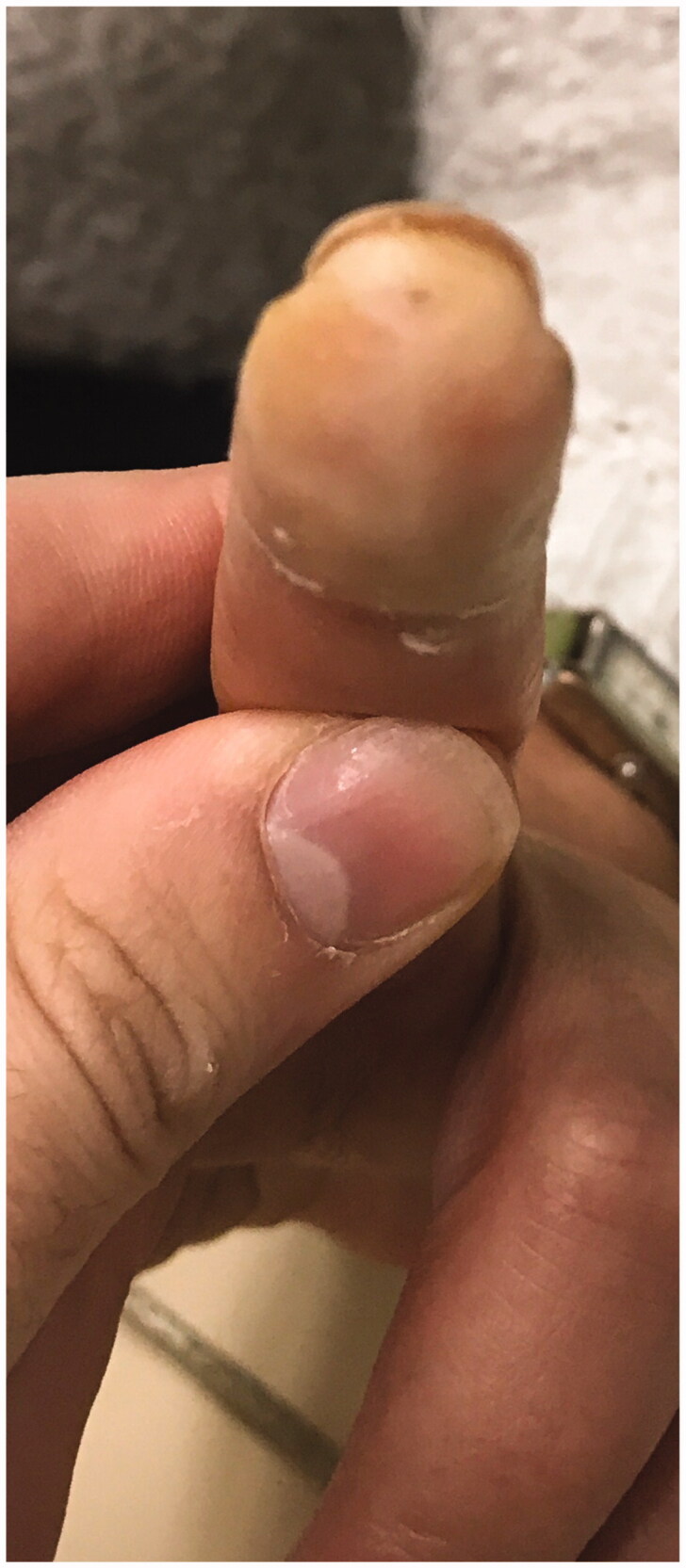
Penetrating injury site (volar).

**Figure 2.**
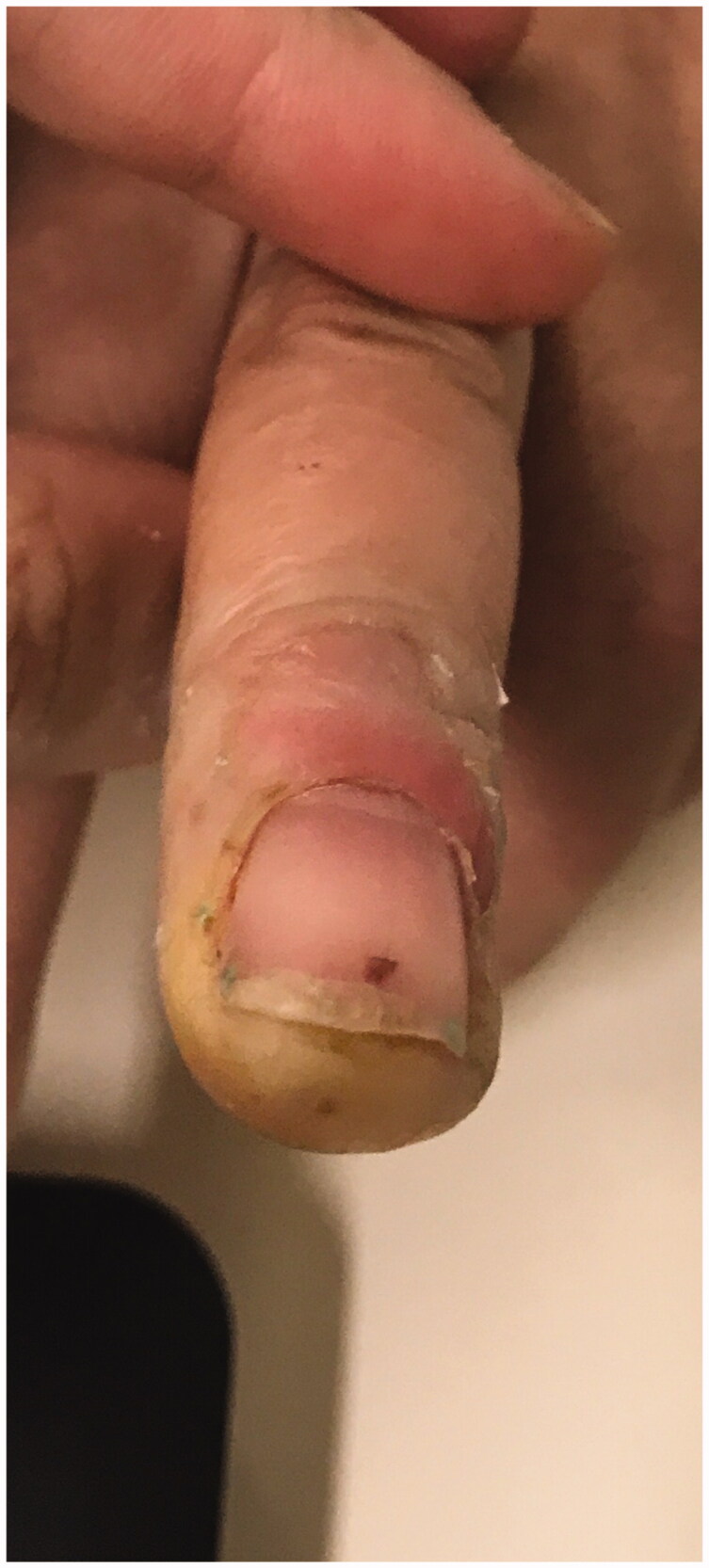
Penetrating injury site (dorsal).

**Figure 3.**
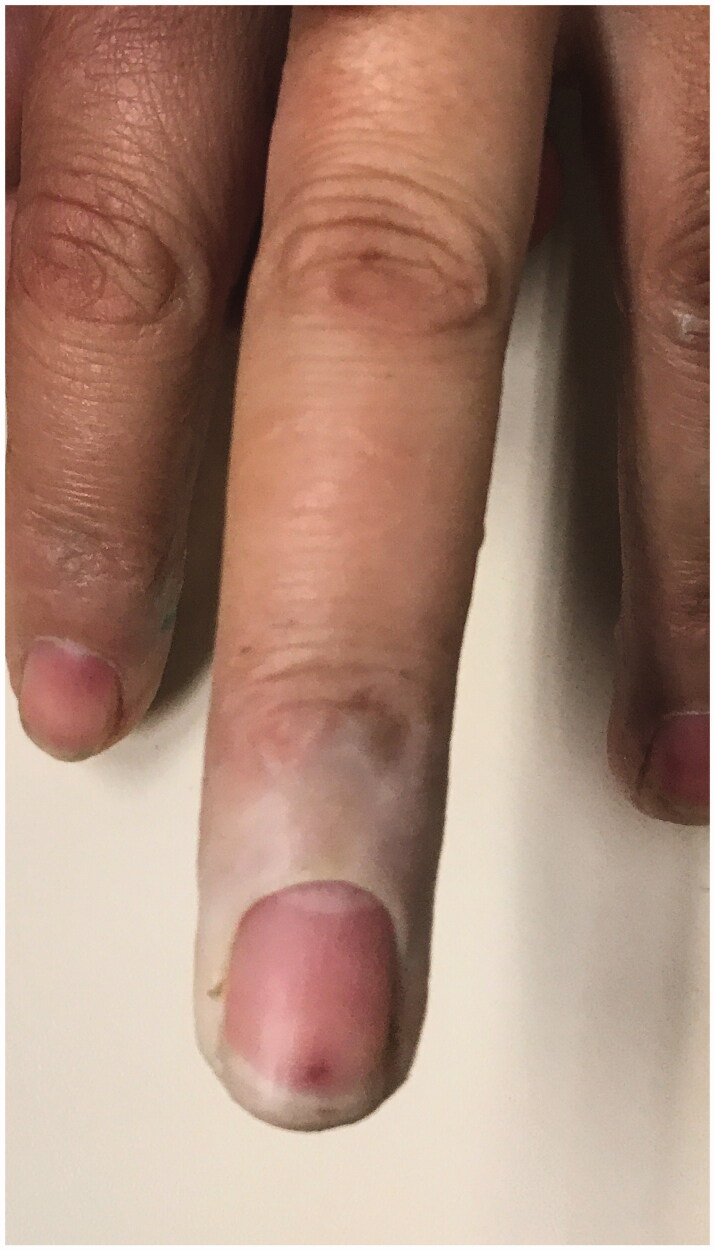
Skin discoloration in the distal third of the right middle finger (dorsal view).

**Figure 4.**
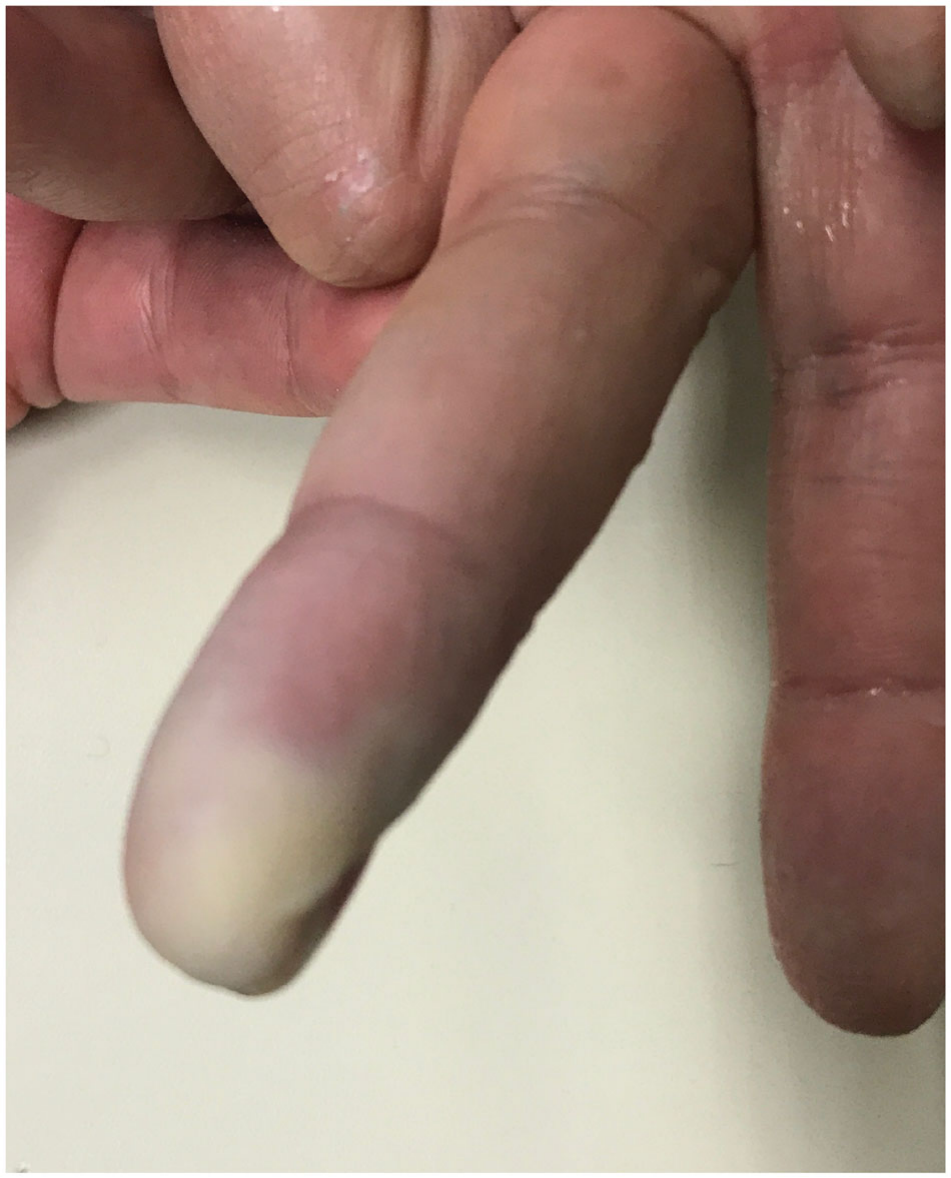
Skin discoloration in the distal third of the right middle finger (volar view).

**Figure 5.**
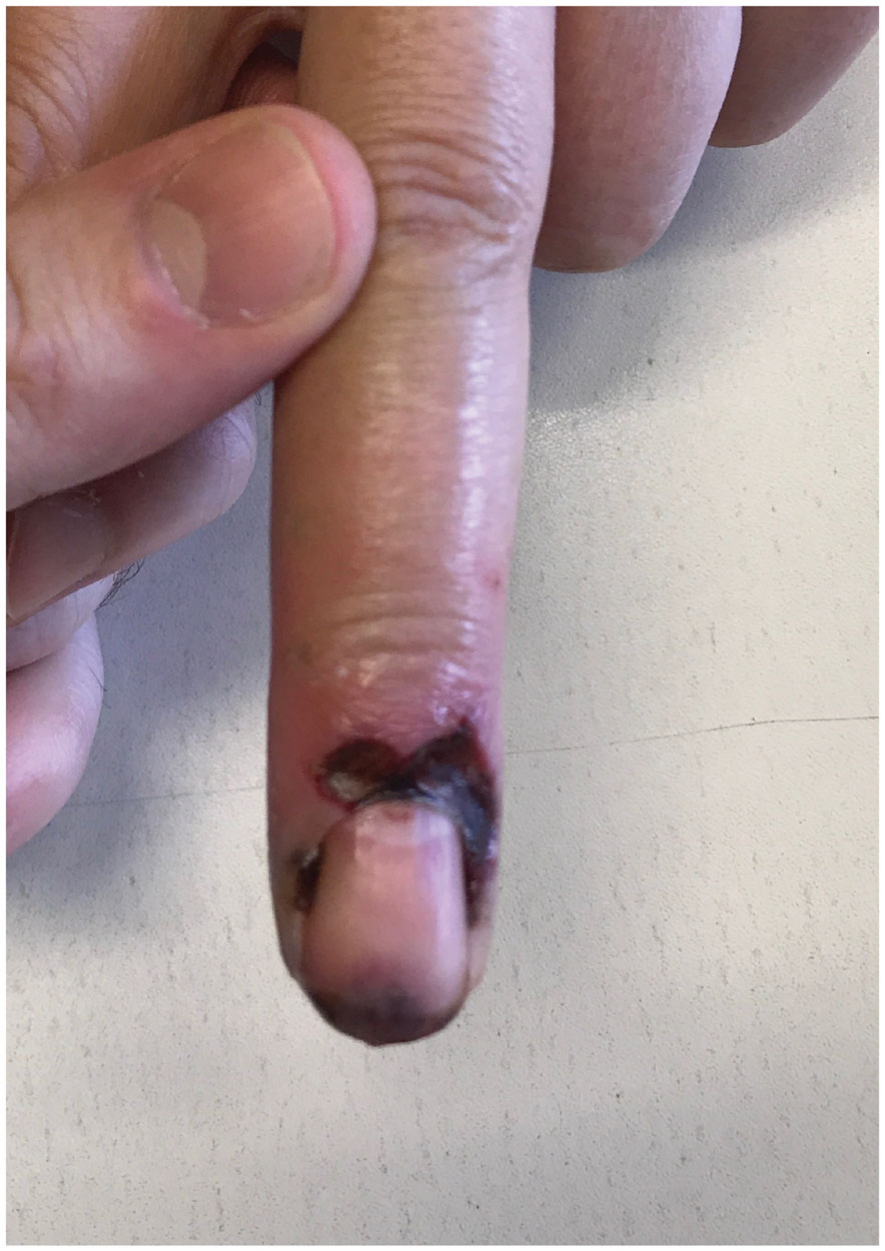
Skin with a hemorrhagic patch.

**Figure 6.**
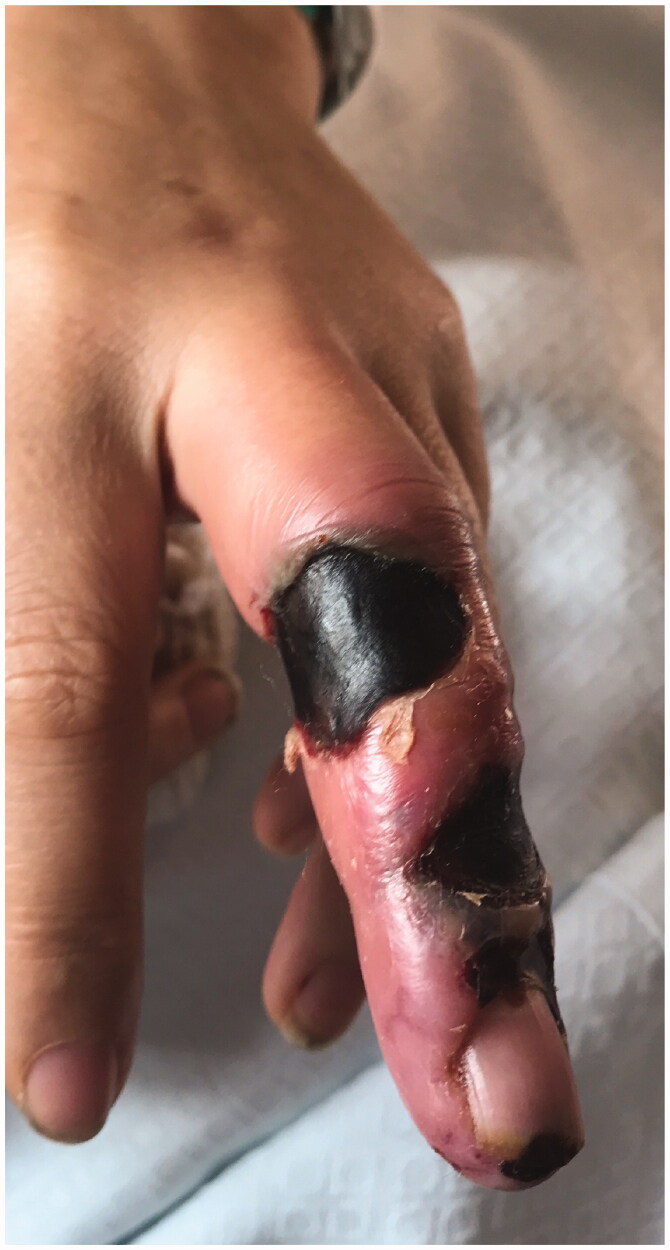
Skin with deep necrosis ulcers (10th day post-trauma).

**Figure 7.**
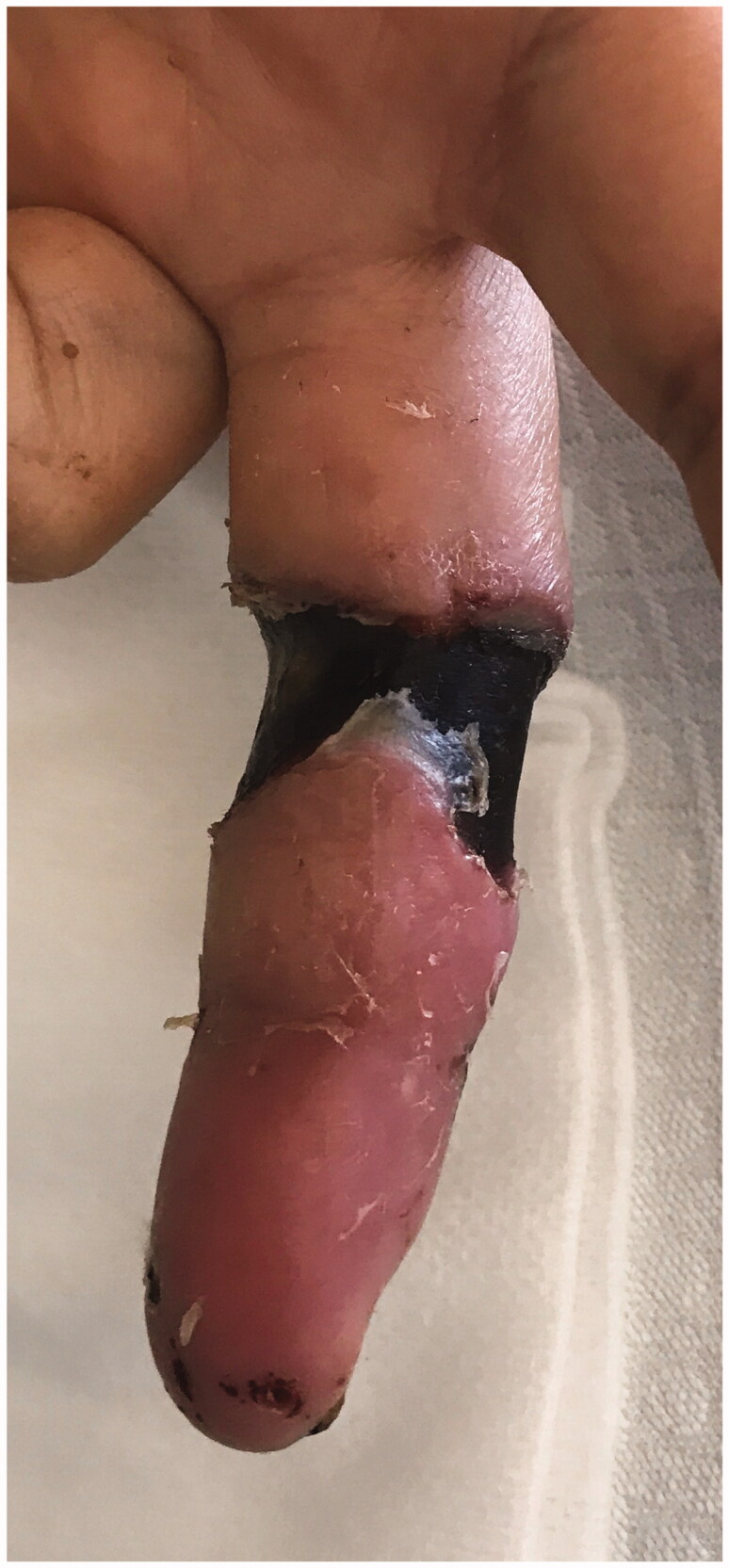
Skin with deep necrosis ulcers (12th day post-trauma - volar view).

**Figure 8.**
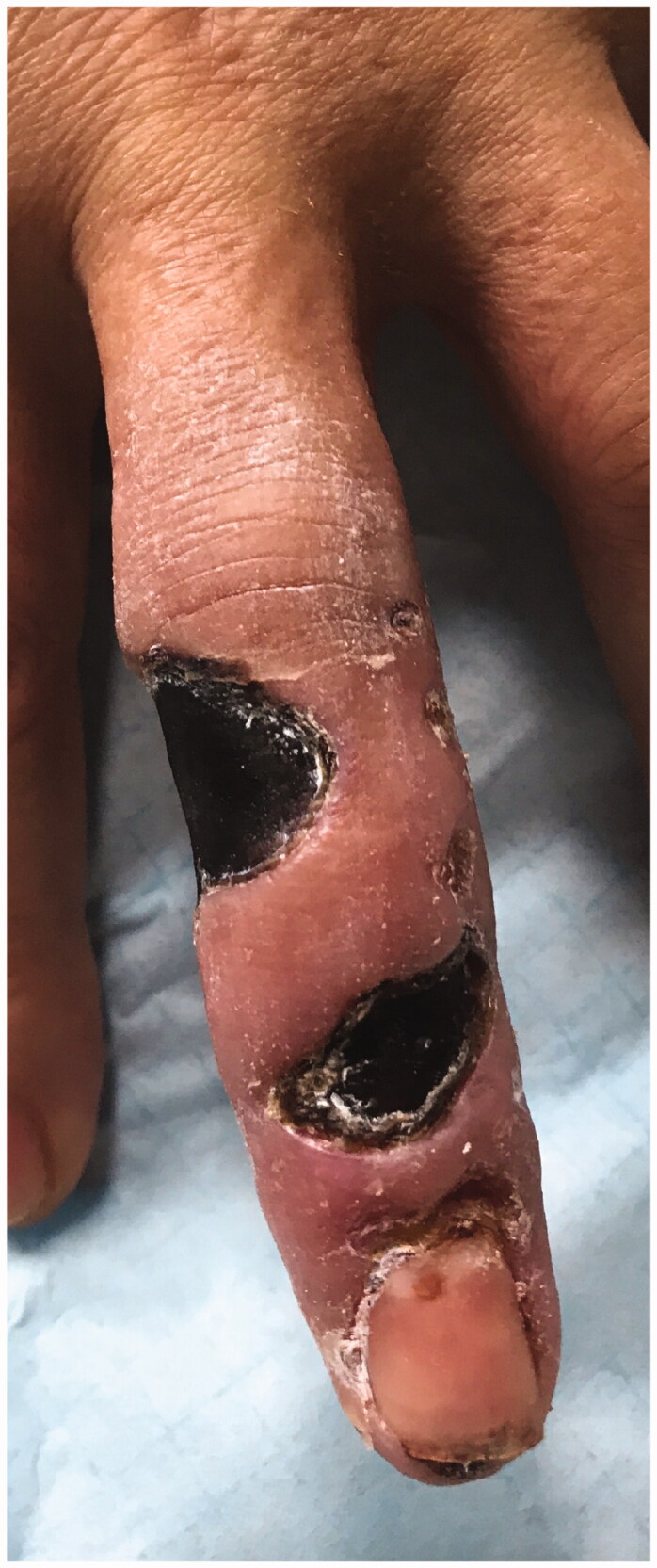
Skin with deep necrosis ulcers (12th day post trauma - dorsal view).

We report a 54-year-old Portuguese woman, healthy, with no know pathological history as diabetes or vasculitis, who presented to our trauma section with skin discoloration in the distal third of her right middle finger after a traumatic penetrating injury with a sewing needle (Figures 1–4). There were no signs of infection at the first observation. On the third day post-trauma, her skin turned dark purple with a hemorrhagic patch (Figure 5). By the 10th day post-trauma, the erythematic area had decreased, but most of the darkly colored skin had progressively turned black with deep necrosis ulcers (Figures 6–8). The application of a cold compress during this period of time on a private clinic apparently aggravated the symptoms.

She returned to our Hospital and was hospitalized. She was administered an intravenous injection of Hydrocortisone 100 mg stat, enoxaparin 40 units subcutaneously every 24 h and Antibiotic (Amoxicillin/Clavulanic acid) for 8 days, taking into account the high probability of secondary infection associated with tissue necrosis. During admission, the signs and symptoms improved gradually. She was discharged with regular visits for follow up.

Other cutaneous and systemic examinations were normal. There was no regional or generalized lymphadenopathy. A skin biopsy was performed and showed necrotic changes caused by ischemia.

A complete blood count, including bleeding and clotting time, and urine examinations were normal. Her chest X-ray, echocardiogram, blood urea, serum creatinine, liver function tests and creatine kinase, were normal. The results of her Venereal Disease Research Laboratory (VDRL) and human immuno- deficiency virus (HIV)-1 and HIV-2 tests were negative.

**Figure 9.**
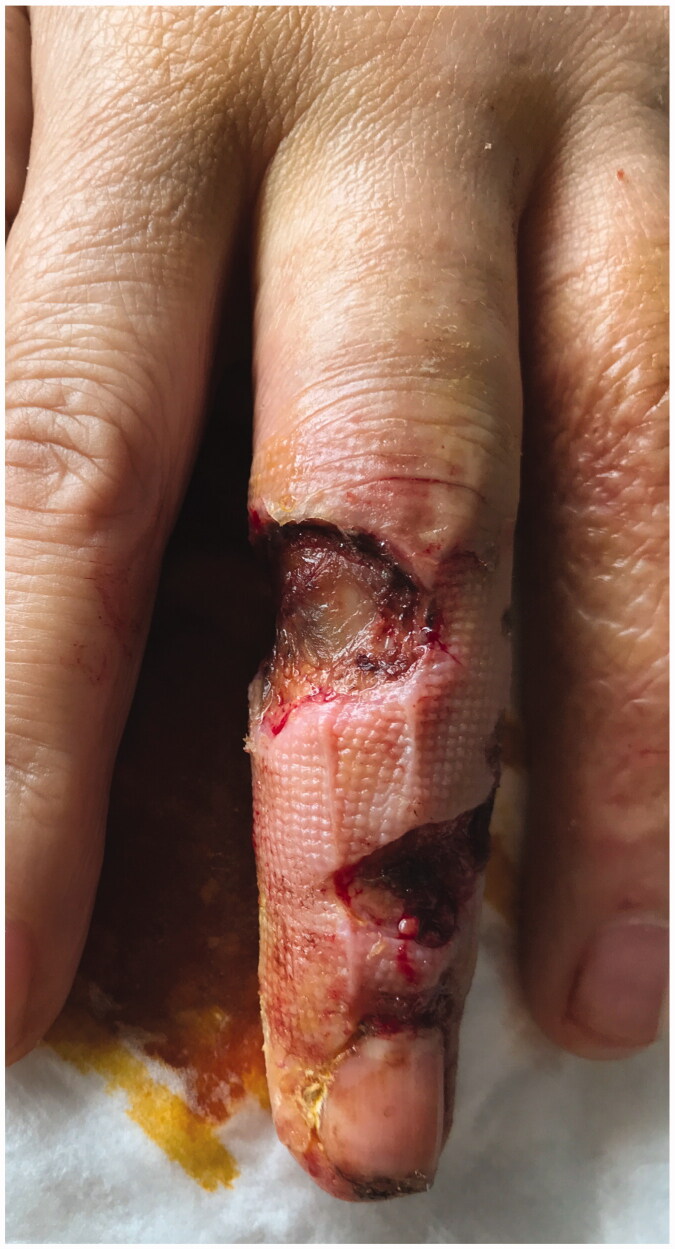
Skin after sucessive surgical debridements and sterile dressings (5 weeks).

**Figure 10.**
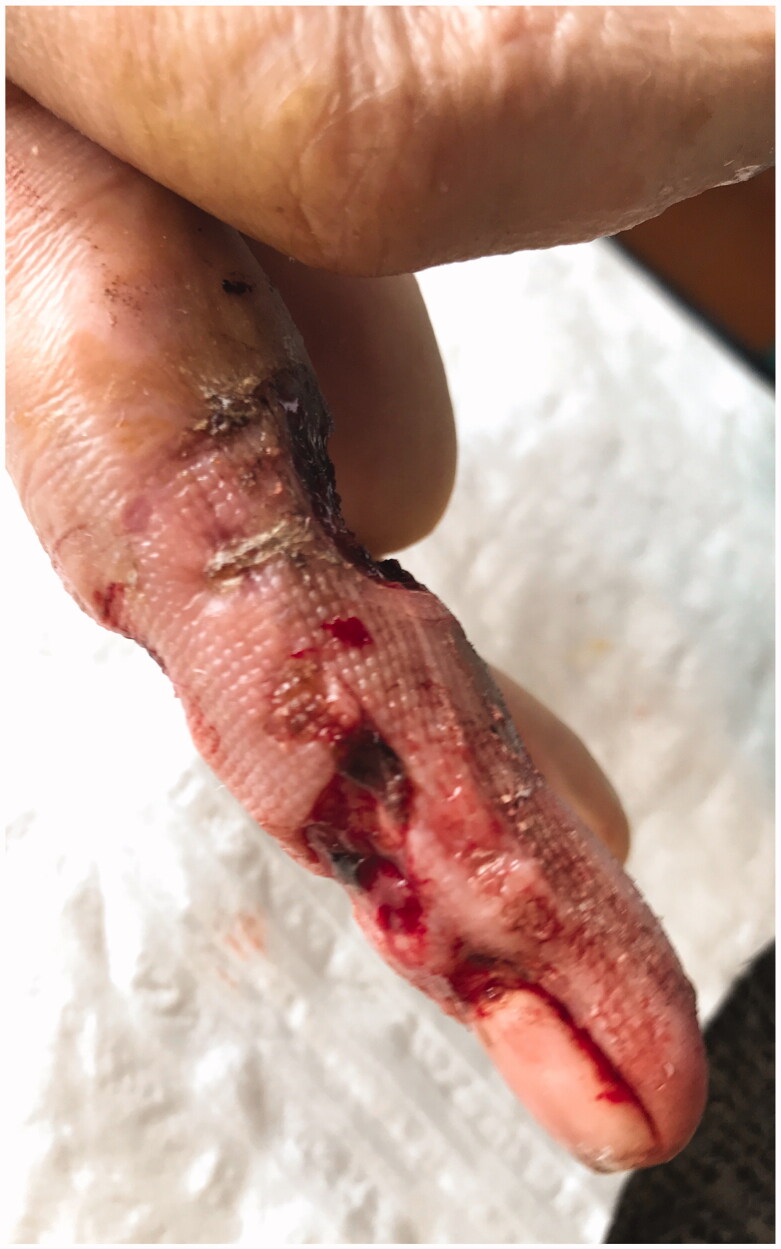
Skin after sucessive surgical debridements and sterile dressings (7 weeks).

**Figure 11.**
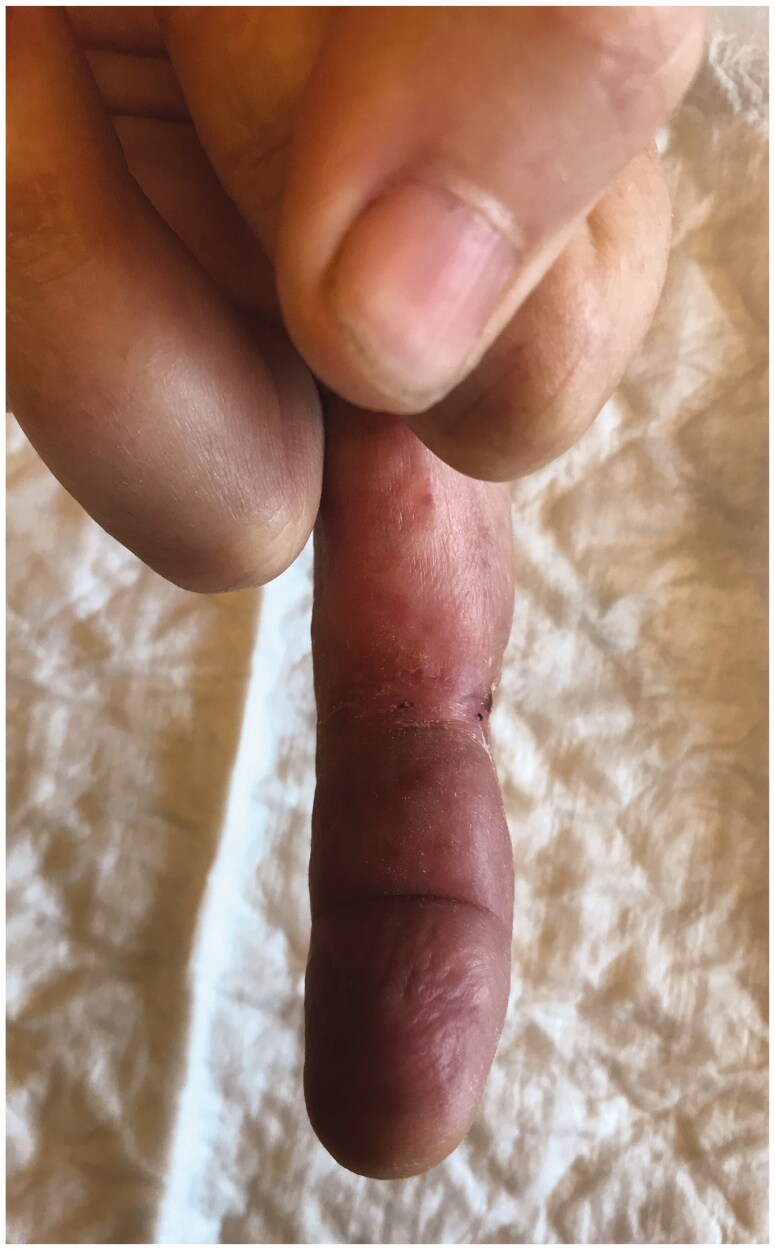
Skin appearence after 15 weeks of treatment.

**Figure 12.**
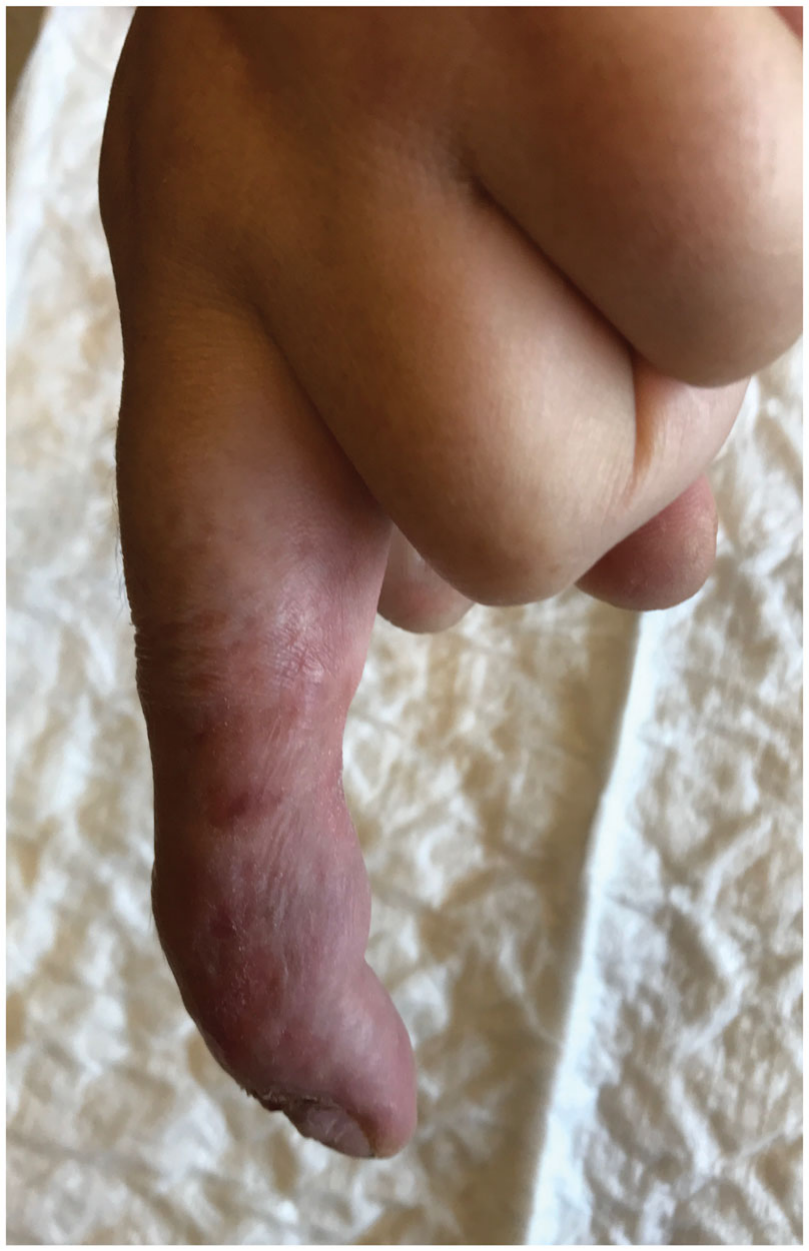
Skin appearence after 16 weeks of treatment.

**Figure 13.**
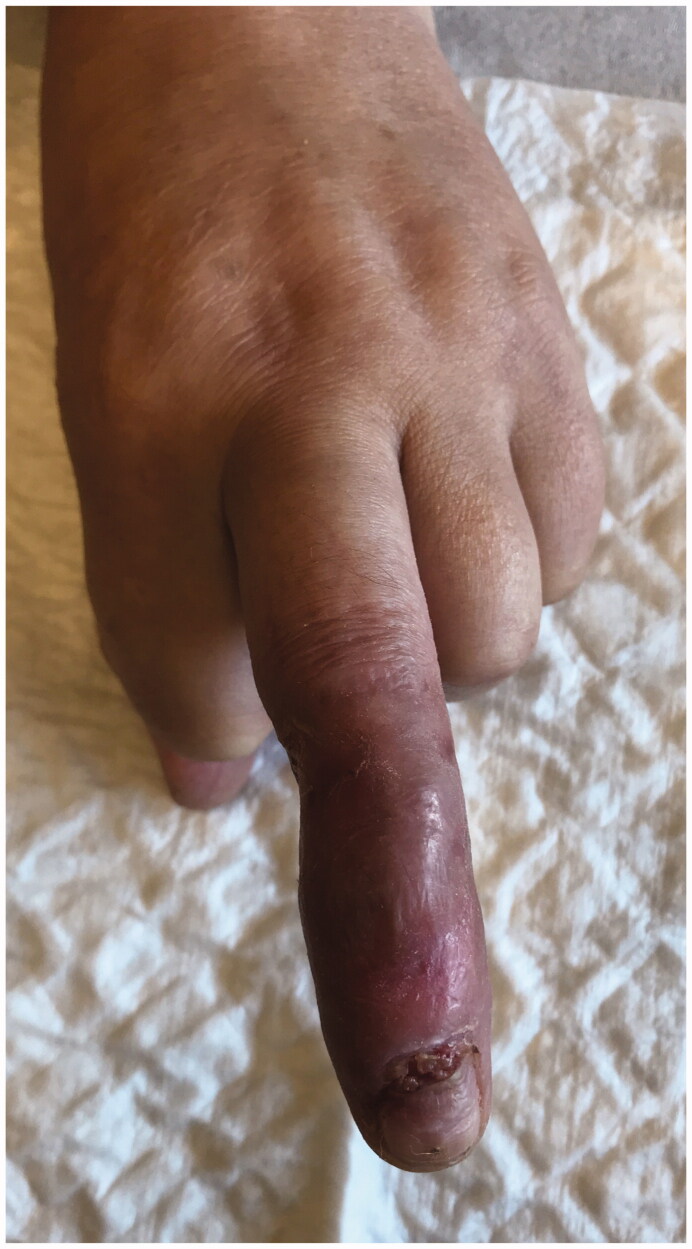
The ulcer healed completely within 18 weeks with scarring.

She was treated with sucessive surgical debridements, sterile dressings three times a week , and analgesics (Figures 9 and 10). The ulcer healed completely within 18 weeks with scarring. (Figures 11–13). In terms of function, the patient maintains slight distal paresthesias, with sequelae joint stiffness, especially in the distal interphalangeal region. Skin graft or reconstructive surgery were not necessary.

## Discussion

Typically Nicolau syndrome presents with pallor, due to a local reflex vasospasm, and pain, rapidly followed by erythema, hemorrhagic patch, blistering, and variable degree of necrosis [4]. This syndrome is more frequent in the pediatric population, mainly in children who are younger than 3 years, in which the phenomenon of artery embolism may be more likely to happen due to the smaller size of the vascular segments involved [5].

The pathogenesis of the disease is not known, but there have been several hypotheses such as stimulation of the sympathetic nerve causing vasospasms and leading to ischemia, embolic occlusions, perivascular inflammation from a cytotoxic reaction to the drugs or induced physical occlusions [6].

One important clinical element is the sudden onset with relation to the injection or trauma, like needle penetration. Necrosis usually comes after hyperemia, discoloration of skin, formation of hemorrhagic patch at the site of injection, and livedoid dermatitis. Local vasospasm causes pallor. One-third of the patients may experience neurologic complications (usually transitional) which are most frequently hypoesthesia and paraplegia.

Diagnosis is mainly clinical. A skin biopsy shows necrotic changes caused by ischemia as we presented before [7]. Initial differential diagnosis include a local toxic reaction to drugs or acute compartment syndrome. Also the differential diagnosis includes vasculitis, fat embolism, left atrial myxomas and Hoigne syndrome. Misdiagnosis of cellulitis lead to use antibiotics as the main treatment and it may be responsible for the failure of treatment for NS. Suspicious malignancy has to be checked by surgical extirpation of the plaque of the lesion and biopsy for macroscopic examination. In our case the needle perforation trauma caused the immediate onset of symptoms.

It is important to identify the stage of the injury to adjust the treatment. There are three distinct stages: in the initial phase, because of severe pain, conservative pain control with analgesics and dressings is usually recommended. Ice pack application increases the acute focal vasospasm and can aggravate the disaster. Systemic antibiotics are suggested. Differential diagnoses are essentially relevant at this stage. In the acute phase, hypothesis of vascular origin and inflammatory sequelae is most reasonable. For this reason systemic steroid and anticoagulant agent are usually used. Warm intermittent compression is also recommended. Finally in the necrotic phase, the patients with NS needs surgical debridement of the affected skin, subcutaneous tissue and muscle in case of clinical and radiographic evidence of tissue necrosis. Skin graft or reconstructive surgery might be necessary [3,8].

When this syndrome is suspected, it is essential to start prophylactic antibiotics, taking into account the high probability of secondary infection associated with tissue necrosis. In some series, secondary staphylococcal infections have been successfully treated with early antibiotic initiation. However, the correct diagnosis is essential because starting antibiotics without knowing the etiology may lead to incomplete treatment and with worse clinical results.

We present an unique case of a NS caused by a sewing needle perfuration trauma with photographic records from the first day. We believe that the traumatic penetration caused local vasospasm and embolic occlusions leading to severe necrotic plates. The application of a cold compress was considered to be an aggravating factor in our patient.

## Conclusions

As the exact etiopathogenesis of this syndrome is not known, there is no standard guidelines for its management. Wound debridement in early stages, systemic antibiotics, and corrective plastic surgery in late stages are essential points for treatment. Clinical differentiation alone is difficult to make a diagnosis of NS and the imaging and laboratory tests assist in the clinical decision.

In our opinion, the success of the treatment was due to the persistence in dressing care and surgical debridement.
